# Clinicians’ Perceptions of an Artificial Intelligence–Based Blood Utilization Calculator: Qualitative Exploratory Study

**DOI:** 10.2196/38411

**Published:** 2022-10-31

**Authors:** Avishek Choudhury, Onur Asan, Joshua E Medow

**Affiliations:** 1 Industrial and Management Systems Engineering Benjamin M Statler College of Engineering and Mineral Resources West Virginia University Morgantown, WV United States; 2 Systems Engineering School of Systems and Enterprises Stevens Institute of Technology Hoboken, NJ United States; 3 Neurocritical Care Neurosurgery, Pathology, and Biomedical Engineering University of Wisconsin School of Medicine and Public Health Madison, WI United States

**Keywords:** artificial intelligence, human factors, decision-making, blood transfusion, technology acceptance, complications, prevention, decision support, transfusion overload, risk, support, perception, safety, usability

## Abstract

**Background:**

According to the US Food and Drug Administration Center for Biologics Evaluation and Research, health care systems have been experiencing blood transfusion overuse. To minimize the overuse of blood product transfusions, a proprietary artificial intelligence (AI)–based blood utilization calculator (BUC) was developed and integrated into a US hospital’s electronic health record. Despite the promising performance of the BUC, this technology remains underused in the clinical setting.

**Objective:**

This study aims to explore how clinicians perceived this AI-based decision support system and, consequently, understand the factors hindering BUC use.

**Methods:**

We interviewed 10 clinicians (BUC users) until the data saturation point was reached. The interviews were conducted over a web-based platform and were recorded. The audiovisual recordings were then anonymously transcribed verbatim. We used an inductive-deductive thematic analysis to analyze the transcripts, which involved applying predetermined themes to the data (deductive) and consecutively identifying new themes as they emerged in the data (inductive).

**Results:**

We identified the following two themes: (1) workload and usability and (2) clinical decision-making. Clinicians acknowledged the ease of use and usefulness of the BUC for the general inpatient population. The clinicians also found the BUC to be useful in making decisions related to blood transfusion. However, some clinicians found the technology to be confusing due to inconsistent automation across different blood work processes.

**Conclusions:**

This study highlights that analytical efficacy alone does not ensure technology use or acceptance. The overall system’s design, user perception, and users’ knowledge of the technology are equally important and necessary (limitations, functionality, purpose, and scope). Therefore, the effective integration of AI-based decision support systems, such as the BUC, mandates multidisciplinary engagement, ensuring the adequate initial and recurrent training of AI users while maintaining high analytical efficacy and validity. As a final takeaway, the design of AI systems that are made to perform specific tasks must be self-explanatory, so that the users can easily understand how and when to use the technology. Using any technology on a population for whom it was not initially designed will hinder user perception and the technology’s use.

## Introduction

### Blood Transfusion and Challenges

Blood product transfusion (BT) is a critical aspect of routine clinical practice, and over 10.5 million units of blood are transfused annually in hospitals within the United States [1,2]. BT is essential across multiple health care domains [3]. There exists a substantial need for blood, and this need has increased, as the burden of chronic diseases has overlapped with increasing life expectancy [4]. Unfortunately, health care systems have been experiencing BT overuse (unnecessary transfusion), that is, patients are being given more blood than what is physiologically required. The practice of transfusion overuse has been a concern in multiple other countries, including the United Kingdom, Spain, Northern Ireland, and South Africa [5-10]. Transfusion overuse can make patients prone not only to immunological reactions, including hemolysis and acute lung injury, but also to circulatory volume overuse and acute heart failure [11]. In 2011, there were 30 casualties reported among transfusion recipients in the United States, and among all associated risks, transfusion-related acute lung injury and volume overload have been significant causes of morbidity [12,13]. Besides health risks, transfusion overuse also contributes to increased hospital expenses and worsens already limited blood product supplies, resulting in shortages.

The drawbacks of transfusion overuse have been long identified by authorities and have instigated much interest in institution-based and national patient blood management initiatives within the United States [11,12,14,15]. Additionally, efforts have been entrusted with clinical studies aiming to optimize blood transfusion practices. Research has proposed clinical practice guidelines and processes to standardize blood transfusion. However, noticeable variability in transfusion practices and related outcomes for patients remains. Deciding to transfuse a patient is not always straightforward or linear, and this decision cannot be consistently made based on specific criteria [12,16]. The determinants of standardized blood transfusion encompass several variables, including the clinical scenario, patient risk factors, comorbidities, vital signs, the rate of anemia onset [17], the bleeding rate, and many others. No one numerical laboratory value can be used as a definitive guide for blood transfusion [12,16]. Other factors, such as an insufficient understanding of transfusion guidelines and the diverse recommendations of medical societies, can also contribute to inconsistencies in blood transfusion practices.

### Blood Utilization Calculation

**Figure 1:**
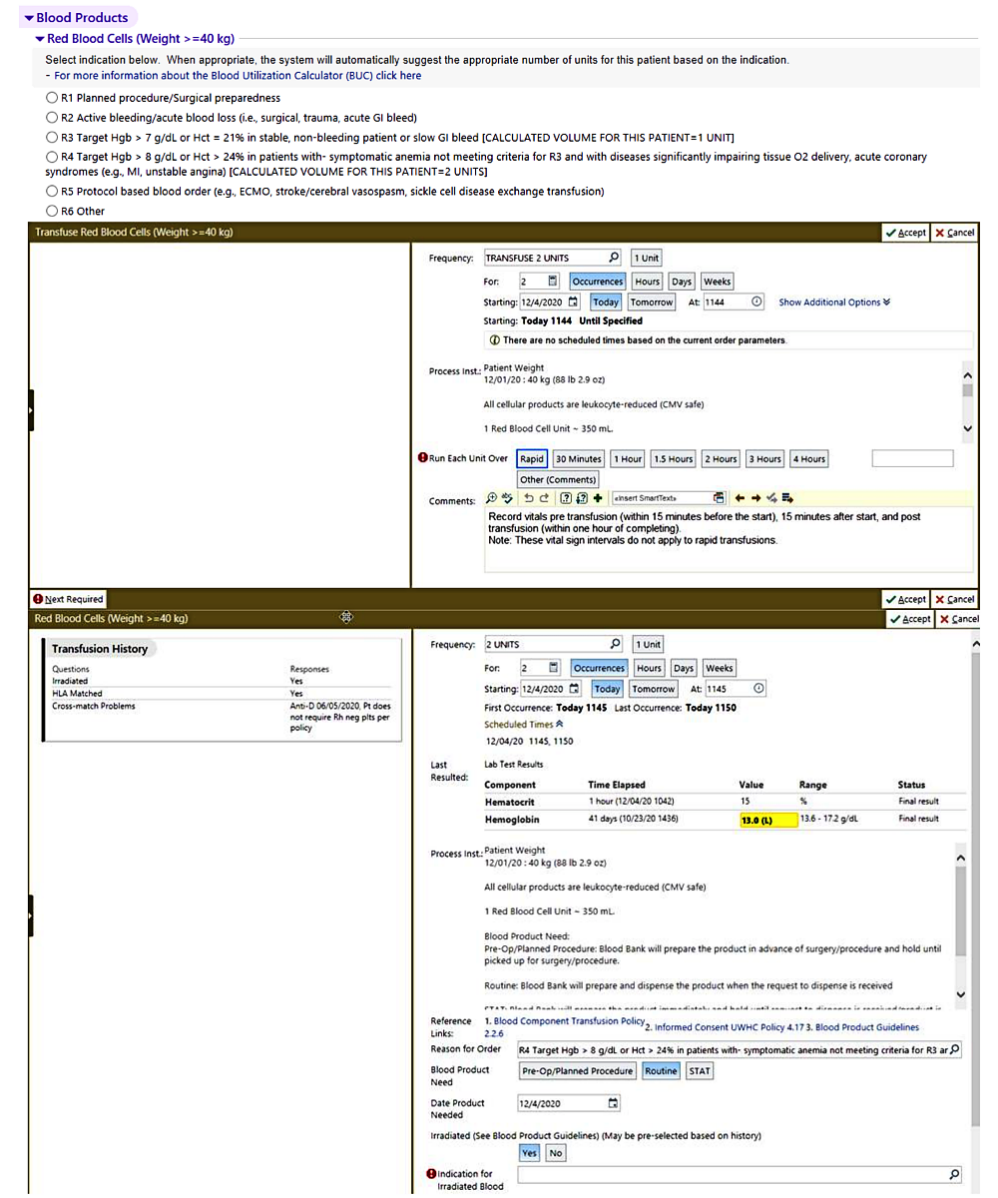
The artificial intelligence–based blood utilization calculator.

To minimize transfusion overuse, a proprietary artificial intelligence (AI)–based blood utilization calculator (BUC) was developed and integrated into the electronic health record at a university hospital in Wisconsin. It is a module of an electronic decision support program known as the Digital Intern (Integrated Vital Medical Dynamics, LLC), and it was designed to ensure the standardization of red blood cell transfusion, following the blood transfusion guidelines. This digital technology runs on a proprietary AI algorithm that provides clinical recommendations for the number of packed red blood cells required to achieve the target hemoglobin or hematocrit value for a given adult patient. It has been reported that the target hemoglobin value was achieved in more than 96% of prescribed transfusions with the help of the BUC (Figure 1) [6,18,19]. It has also been pointed out that the BUC is more consistent than clinicians [18]. Further details of the BUC have been explained elsewhere [20].

Despite its promising performance, the BUC remains underused in the clinical setting. Clinicians often reject BUC recommendations [19], resulting in transfusion overuse and related expenses. Therefore, this qualitative study aims to explore how clinicians perceived this AI-based decision support system and, consequently, understand the factors hindering BUC use.

## Methods

### Ethics Approval

This study took place in collaboration with a university hospital in Wisconsin. It obtained ethical approval from the institutional review board of the Stevens Institute of Technology, Hoboken (institutional review board ID: 2022-021N).

### Semistructured Interviews

This study used a qualitative analysis of semistructured interviews to explore the factors affecting clinicians’ decision-making regarding blood transfusion. Table 1 shows the interview guide. The fundamental principle of qualitative interviewing is to provide a framework for participants to express their understanding on their terms [21]. Semistructured interviews are typically used in qualitative research and are among the most common data sources in health care research [22]. They consist of several key questions that not only help define the areas to be explored but also allow interviewers or interviewees to diverge from pursuing an idea or response in more detail [23]. Most importantly, the flexibility of this approach (in comparison to structured or unstructured interviews) enables interviewers to stay focused on their research agenda and allows for the discovery or elaboration of information that is important to participants but may not have previously been thought of as pertinent by the research team [23].

**Table 1:** Interview guide.

Topic	Guiding questions	Possible follow-up questions
General experience with the BUC^a^	“I am curious to know how you feel when using BUC”	“How did it impact your clinical performance?” “How does using BUC impact your decision-making?”
General experience with the BUC	“What are your thoughts about the impact of BUC on patient safety”	“Under what conditions do you think BUC can cause harm to the patient or give the wrong recommendation?” “Can you please share your experience when BUC helped you perform better?” “Did it ever happen when BUC helped you correct or negatively impacted your decision – can you elaborate on that with an example”
Workload	“Clinicians are often overloaded with work. How do you feel BUC has helped reduce or increase some of your workloads?”	“Can you give an example when BUC made things easier, which otherwise would require more work” “Can you give an example when BUC made things difficult or confusing that otherwise would be easy”
Decision-making	“When you give the final recommendation about the number of blood units to be transfused for a patient – how do you know when to go with the BUC recommendation and when to make your judgment?”	“Can you elaborate on how you make a judgment when the BUC recommendation contradicts your decision?” “Have you ever changed your decision after looking at the BUC recommendation? Can you please elaborate?”
Closure	“Thank you for taking the time to share your BUC experience. Is there anything else you think I should know?”	N/A^b^

^a^BUC: blood utilization calculator.

^b^N/A: not applicable.

### Data Collection and Analysis

We interviewed 10 clinicians (BUC users) until the data saturation point was reached [24]. The saturation sampling method is a well-known methodological principle in qualitative research. It is used to determine, based on the data that have been collected and analyzed, whether further data collection is unnecessary [24]. We decided to stop recruitment after the 10th interview, as we attained thematic saturation. Moreover, a high degree of consensus had begun to emerge among the clinicians who were interviewed, and the information retrieved was sufficient for satisfying the aims of this investigation. According to the literature, data saturation can be reached with 9 to 17 interviews [25].

The interviews were conducted over a web-based platform and were recorded. The audiovisual recordings were then anonymously transcribed verbatim. Each participant was given a US $50 gift card for completing the interview. Each interview lasted approximately 20 to 30 minutes. We used an inductive-deductive thematic analysis to analyze the transcripts [26], which involved applying predetermined themes to the data (deductive) and consecutively identifying new themes as they emerged in the data (inductive) [27]. This method included the interpretation of the text and an analysis of what the text discussed, specifically identifying work system elements and cognitive human factors that influenced clinicians’ use of the BUC and clinical decision-making. We also prepared the COREQ (Consolidated Criteria for Reporting Qualitative Research) checklist (Multimedia Appendix 1).

## Results

### Overview of Clinicians’ Perceptions

A total of 10 clinicians from different clinical departments participated in this study. All of the participants were frequent users of the BUC (at least once per week) and had used the technology for at least 1 year. As shown in Textbox 1, we identified the following two themes: (1) workload and usability and (2) clinical decision-making. We discussed each theme briefly by providing detailed quotations.

**Clinicians’ perceptions of the artificial intelligence–based blood utilization calculator (BUC).**

**Workload and usability**

- Sample quotes reflecting a negative perception
  “I remember the first time I saw it, like sort of reading through a lot of options, like, which one of these do apply to me so that I can get the transfusion order to go through, which can take a little bit of time.”
  “It requires quite a few extra clicks to go through the other indication to get it, to let you give plasma for this indication that is often recommended by hepatology.”
  “BUC slowed me down whenever I had to figure out how to bypass the BUC system to get the threshold, we knew we needed for that particular patient.”
  “I think there is a risk of getting down in the cognitive fatigue of decision-making and figuring out which box to click.”
- Sample quotes reflecting a positive perception
  “Earlier, we used to decide how many units like haphazardly, but with BUC, I like that it does part of my thinking. Well, I would say it’s easier because now I don’t have to think as it tells me how many units of blood, I need to give a patient.”
  “Overall, I like using it because it takes a lot of the thinking out of it in terms of calculations and stuff. BUC makes it a lot easier for every standard patient care.”
  “I think it’s pretty easy to use. It’s straightforward. It just kind of like leads you exactly through the process.”
  “I find it relatively easy to work with, and I like it because it’s straightforward. I just choose whatever I want, and it calculates or puts in the numbers.”
  “I think it’s user-friendly and easy. I don’t think it adds extra work. I’ve used it in general surgery trauma, like when you’re doing more complicated, like a resuscitation, like one-to-one to one ratio and, um, I think it was pretty user-friendly for that as well.”

**Clinical decision-making**

- Sample quotes reflecting a negative perception
  “BUC does not improve my decision-making. It’s not groundbreaking in any way. It’s just more of a reminder of what I have already been taught as a young physician.”
- Sample quotes reflecting a positive perception
  “Overall, it helps you, and I think it helps, you know, determine how much blood a patient need. Kind of making sure we’re ordering blood for the right patient indication, reminding us of appropriate criteria. So, I think overall; I think it’s a pretty useful tool that we use.”
  “I think it’s helpful because it explains like hemoglobin of several patients. If a patient has low platelets, you might have a higher hemoglobin goal. Um, so it’s nice to have that spelled out for you, so you don’t have to look it up elsewhere and then come back and make the decisions.”
  “I like having the guidelines built-in so that you know when you’re doing something that is, um, the, that is the guideline or evidence based. And, you know, when you are deviating from that and therefore hopefully have a good reason for it and are at least cognizant of the fact that you're deviating.”
  “I remember, yeah, a couple of times where we initially wanted to give like two packets of blood, but then [BUC] recommended only one, and we kind of went back and we’re like, well, I think the tool is right. Like, we only need to give one unit of blood in this case.”
  “If BUC is telling me that I’m ordering too much blood, I go back, thinking, okay, does the patient need this much blood? So, it’s more like I’m ensuring I follow the standard of care, except for those exceptional patient circumstances.”

### Workload and Usability

For standard care patients, BUC use often helps to standardize blood transfusion and minimize cognitive workload. Overall, clinicians found the BUC to be user-friendly and intuitive. They acknowledged that the BUC has an easy learning curve. Clinicians attending patients with trauma found the BUC user-friendly. However, the perceptions of BUC-related workload were not consistent across all users. According to some clinicians, the BUC was an add-on to their clinical work; they found the BUC interface to be complex for new users. A clinician noted that the interface of the BUC can result in confusion and incorrect transfusion dosages due to inconsistent automation across different blood work processes.

### Clinicians’ Decision-making and BUC Performance

Clinicians found the BUC to be a helpful technology that often assisted them in making informed clinical decisions regarding blood transfusion, but it did not necessarily improve their decision-making. By providing necessary information regarding transfusion goals, the BUC helped clinicians make faster decisions. They acknowledged the benefits of having the BUC, which enabled them to adhere to the transfusion guidelines. It encouraged them to think critically about their patients and BT practices. Another critical finding was how clinicians made transfusion decisions when their intuition contradicted the BUC. Clinicians said that they consulted their seniors or followed their judgments whenever their decisions failed to match the BUC recommendations. In other words, clinicians typically trusted the BUC when its recommendations matched their assessments or when a patient had a very standard clinical status, no health complications, or no notable health history. Clinicians also acknowledged that they bypassed BUC recommendations when the recommendations did not match their judgments. Nevertheless, in a few instances, clinicians considered BUC recommendations and changed their judgments after revisiting the patient’s health status. In some other cases, the BUC encouraged discussion among clinicians and provided them with an opportunity to adhere to transfusion guidelines.

## Discussion

The importance of human factors and AI in health care has been well established by several studies and reputed authorities across all significant health care establishments. This is the first study to explore clinicians’ perceptions of an AI-based BUC (ie, an AI decision support system).

### Workload and Usability

Clinicians have limited time in their visits and are often overloaded with the burden of clinical documentation. Integrating user-friendly, AI-based decision support systems can effectively assist clinicians and reduce their workloads. Developing a user-friendly and safe technology mandates human factors consideration. Human factors enable us to understand the importance of users’ needs and how they may vary based on users’ expertise, their environment, and the sensitivity of patients. In our study, depending on their clinical expertise and the patient type, different clinicians perceived the BUC differently. Some found the BUC useful, while others perceived it to be confusing and hard to use, since the technology was not tailored to their needs.

Certain users were not sure when to use and when not to use this technology and oftentimes used the BUC for situations that were beyond its scope (eg, on pediatric patients or patients with sickle cell disease). These users developed a negative perception of the BUC because it was not performing as per their expectations. The BUC is not designed for patients with internal bleeding or sickle cell disease or for ordering blood for scheduled surgeries. It was only built to analyze a given blood value and recommend a transfusion volume to help clinicians achieve their self-selected target blood level. However, trying to use the BUC on other patient types or for other purposes, at times, negatively influenced users’ perceptions of the technology. Clinicians often had to figure out a way to bypass the system and place their blood transfusion order, adding to their existing workloads and slowing down the transfusion process. Nevertheless, when the BUC was used on the appropriate patient population, clinicians found it user-friendly and acknowledged that the technology helped reduce their cognitive workloads and, overall, assisted them with their BT tasks and related decision-making.

User-centered design, wherein the user is centrally involved in all phases of the design process, is essential for AI health care technologies. However, designing user-friendly technologies becomes challenging when the user environments and activities are varied (eg, uncrossed transfusion, massive transfusion, etc). This study shows that usability issues can worsen due to the heterogeneity of applications, users’ needs, and how users use the technology. The unclear design of AI technologies can result in added workloads; increase the likelihood of patient harm; and, most importantly, hinder clinicians’ intent to use the technology. Therefore, adequate training and clarification on the scope, functionality, limitations, and role of a given technology are important for wider acceptance and use.

According to our findings, one way to improve BUC use and acceptance is to have a tailored interface design that automatically detects the treated population based on existing electronic medical record data, the time when a transfusion needs to be ordered (eg, immediately), and the purpose of a transfusion (eg, potential operative need). This approach can ensure that clinicians are shown commonly used information, along with options that are relevant to their patients’ needs at a given moment. A tailored BUC design would also ensure selective situation awareness. For example, allowing clinicians to concentrate on relevant details about their patients may help them avoid unnecessary working memory use. Additionally, implementing functions that prevent the BUC from being used on patients who do not fall within its scope can help minimize errors and prevent clinicians from developing a negative perception of the technology. This can be achieved either by incorporating an alert system within the BUC that would flag every time a user uses the technology on any patient outside of the target population or by completely disabling the BUC whenever an incorrect patient type is detected.

### Clinical Decision-making

One of this study's main contributions, as well as its novelty, is that it captured the impact of an AI-based decision support system (ie, the BUC) on clinical decision-making. We did not notice any negative impact of the BUC on clinical decision-making. Clinical decision-making is a complex process that necessitates a multidisciplinary systemic approach, encompassing psychology, cognition, and statistics. It is considered a context-driven, time-dependent, and evolving process that requires data collection, interpretation, and evaluation to select the appropriate choice of action [28]. For example, the choice of how much blood should be transfused to a specific patient depends on their body weight, medical status, medical history, rate of blood loss (if any), and treatment plan, among many other factors. Due to such factors, care coordination [29] and shared decision-making in clinical practices are challenging. Our findings indicate the positive impact of the BUC on clinicians' decision-making; the technology acted as an assistive digital platform, promoting well-informed BT. Such impacts of AI have been seen in other fields of medicine [30,31].

In the literature on decision-making, intuitive and analytical decision-making [32] are the two predominant decision-making styles. Intuitive decision-making has been portrayed as an automatic [33] decision process that can be shaped by the work environment and contextual skills [34,35]. Senior clinicians have been observed to prefer the intuitive approach [36]. Their tendency to use the intuitive approach is due to their experience and ability to make faster and more accurate clinical decisions [37]. Our study captured the same tendency, as attending clinicians seldom considered BUC recommendations. Almost in every situation, when the recommendations generated by the BUC contradicted senior clinicians’ judgments, they always followed their judgments, thereby exhibiting confirmation bias.

This study has limitations. It was a single-institution assessment that was conducted within an academic health care establishment. Further, the clinicians who participated were a convenience sample, which introduced self-selection bias. Additionally, the clinicians, per their clinical specialties, were not those who advised or performed blood transfusions the most often. However, a diverse population of clinicians, in terms of clinical expertise, was recruited. Future longitudinal research may help quantify the BUC’s impact on patient safety.

### Conclusion

This study highlights that analytical efficacy alone does not ensure technology use or acceptance. The overall system’s design, user perception, and users’ knowledge of the technology are equally important and necessary (limitations, functionality, purpose, and scope). Therefore, the effective integration of AI-based decision support systems, such as the BUC, mandates multidisciplinary engagement, ensuring the adequate initial and recurrent training of AI users while maintaining high analytical efficacy and validity. As seen in this study, all clinicians had different needs that the BUC did not fully address, and the fact that the system's design was not indicative of its actual purpose or target patient population confused its users and hindered its use in the hospital.

As a final takeaway, an AI technology such as the BUC, if not designed for individual users at the department level, might not be used as intended. The design of such AI systems that are made to perform specific tasks must be self-explanatory, so that the users can easily understand how and when to use the technology. AI technologies in health care are only designed and developed to help clinicians identify patterns they would typically overlook. Nevertheless, if clinicians only consider AI recommendations when such recommendations complement their professional and personal judgments or use AI technology on the wrong population, then the motives for having an AI technology in the first place would be in vain.

## Appendix

Multimedia Appendix 1 COREQ (Consolidated Criteria for Reporting Qualitative Research) checklist.

## Abbreviations

AI: artificial intelligence
BT: blood product transfusion
BUC: blood utilization calculator
COREQ: Consolidated Criteria for Reporting Qualitative Research

## References

[1] Sadana D, Kummangal B, Moghekar A, Banerjee K, Kaur S, Balasubramanian S, Tolich D, Han X, Wang X, Hanane T, Mireles-Cabodevila E, Quraishy N, Duggal A, Krishnan S (2021). Adherence to blood product transfusion guidelines-An observational study of the current transfusion practice in a medical intensive care unit. Transfus Med.

[2] Jones JM, Sapiano MRP, Savinkina AA, Haass KA, Baker ML, Henry RA, Berger JJ, Basavaraju SV (2020). Slowing decline in blood collection and transfusion in the United States - 2017. Transfusion.

[3] Armstrong B (2008). Benefits and risks of transfusion. ISBT Sci Ser.

[4] Bediako AA, Ofosu-Poku R, Druye AA (2021). Safe blood transfusion practices among nurses in a major referral center in Ghana. Adv Hematol.

[5] Adedayo T, O'Mahony D, Adeleke O, Mabunda S (2021). Doctors' practice and attitudes towards red blood cell transfusion at Mthatha Regional Hospital, Eastern Cape, South Africa: A mixed methods study. Afr J Prim Health Care Fam Med.

[6] Connor JP, Cunningham AM, Raife T, Rose WN, Medow JE (2017). Standardization of transfusion practice in organ donors using the Digital Intern, an electronic decision support algorithm. Transfusion.

[7] Joy PJ, Bennet SJ (2012). The appropriateness of blood transfusion following primary total hip replacement. Ann R Coll Surg Engl.

[8] Díaz MQ, Borobia AM, Erce JAG, Maroun-Eid C, Fabra S, Carcas A, Frías J, Muñoz M, USEES-URG Research Group (2017). Appropriate use of red blood cell transfusion in emergency departments: a study in five emergency departments. Blood Transfus.

[9] Barr PJ, Donnelly M, Cardwell CR, Parker M, Morris K, Bailie KEM (2011). The appropriateness of red blood cell use and the extent of overtransfusion: right decision? Right amount?. Transfusion.

[10] Salverda M, Ketharanathan N, Van Dijk M, Beltchev E, Buys H, Numanoglu A, Van As AB (2017). A review of blood transfusions in a trauma unit for young children. S Afr Med J.

[11] Mehta N, Murphy MF, Kaplan L, Levinson W (2021). Reducing unnecessary red blood cell transfusion in hospitalised patients. BMJ.

[12] Goodnough LT, Levy JH, Murphy MF (2013). Concepts of blood transfusion in adults. Lancet.

[13] Vamvakas EC (2013). Reasons for moving toward a patient-centric paradigm of clinical transfusion medicine practice. Transfusion.

[14] American Association of Blood Banks: Five things physicians and patients should question. Choosing Wisely.

[15] Padhi S, Kemmis-Betty S, Rajesh S, Hill J, Murphy MF, Guideline Development Group (2015). Blood transfusion: summary of NICE guidance. BMJ.

[16] Goodnough LT, Audet AM (1996). Utilization review for red cell transfusions. Are we just going through the motions?. Arch Pathol Lab Med.

[17] Goodnough LT, Despotis GJ, Hogue CW Jr, Ferguson TB Jr (1995). On the need for improved transfusion indicators in cardiac surgery. Ann Thorac Surg.

[18] Connor JP, Raife T, Medow JE (2018). Outcomes of red blood cell transfusions prescribed in organ donors by the Digital Intern, an electronic decision support algorithm. Transfusion.

[19] Connor JP, Raife T, Medow JE, Ehlenfeldt BD, Sipsma K (2018). The blood utilization calculator, a target-based electronic decision support algorithm, increases the use of single-unit transfusions in a large academic medical center. Transfusion.

[20] Choudhury A, Asan O, Medow JE (2022). Effect of risk, expectancy, and trust on clinicians' intent to use an artificial intelligence system -- Blood Utilization Calculator. Appl Ergon.

[21] Patton MQ (2002). Two decades of developments in qualitative inquiry: A personal, experiential perspective. Qual Soc Work.

[22] DeJonckheere M, Vaughn LM (2019). Semistructured interviewing in primary care research: a balance of relationship and rigour. Fam Med Community Health.

[23] Pope C, Ziebland S, Mays N (2000). Qualitative research in health care. Analysing qualitative data. BMJ.

[24] Saunders B, Sim J, Kingstone T, Baker S, Waterfield J, Bartlam B, Burroughs H, Jinks C (2018). Saturation in qualitative research: exploring its conceptualization and operationalization. Qual Quant.

[25] Hennink M, Kaiser BN (2022). Sample sizes for saturation in qualitative research: A systematic review of empirical tests. Soc Sci Med.

[26] Braun V, Clarke V (2014). What can "thematic analysis" offer health and wellbeing researchers?. Int J Qual Stud Health Well-being.

[27] Bingham AJ, Witkowsky P, Vanover CF, Mihas PA, Saldana J (2021). Deductive and inductive approaches to qualitative data analysis. Analyzing and Interpreting Qualitative Research: After the Interview.

[28] Tiffen J, Corbridge SJ, Slimmer L (2014). Enhancing clinical decision making: development of a contiguous definition and conceptual framework. J Prof Nurs.

[29] Hepp SL, Suter E, Jackson K, Deutschlander S, Makwarimba E, Jennings J, Birmingham L (2015). Using an interprofessional competency framework to examine collaborative practice. J Interprof Care.

[30] Hötker AM, Da Mutten R, Tiessen A, Konukoglu E, Donati OF (2021). Improving workflow in prostate MRI: AI-based decision-making on biparametric or multiparametric MRI. Insights Imaging.

[31] Yang Q, Steinfeld A, Zimmerman J (2019). Unremarkable AI: Fitting intelligent decision support into critical, clinical decision-making processes.

[32] Falzer PR, Garman DM (2012). Image Theory’s counting rule in clinical decision making: Does it describe how clinicians make patient-specific forecasts?. Judgm Decis Mak.

[33] Custers EJFM (2013). Medical education and cognitive continuum theory: an alternative perspective on medical problem solving and clinical reasoning. Acad Med.

[34] Lockwood C (2011). Clinical judgement and decision-making in nursing and interprofessional healthcare. Int J Evid Based Healthc.

[35] Parker-Tomlin M, Boschen M, Glendon I, Morrissey S (2019). Factors influencing health practitioners' cognitive processing and decision-making style. J Interprof Care.

[36] McLaughlin JE, Cox WC, Williams CR, Shepherd G (2014). Rational and experiential decision-making preferences of third-year student pharmacists. Am J Pharm Educ.

[37] Jung WH, Kim SN, Lee TY, Jang JH, Choi CH, Kang DH, Kwon JS (2013). Exploring the brains of Baduk (Go) experts: gray matter morphometry, resting-state functional connectivity, and graph theoretical analysis. Front Hum Neurosci.

